# The ONE Study compares cell therapy products in organ transplantation: introduction to a review series on suppressive monocyte-derived cells

**DOI:** 10.1186/2047-1440-1-11

**Published:** 2012-09-28

**Authors:** Edward K Geissler

**Affiliations:** 1 Department of Surgery, University Hospital Regensburg, University of Regensburg, Regensburg, Franz-Josef-Strauss-Allee 11, 93053, Regensburg, Germany

## The problem

Organ transplantation has evolved into a reliable life-saving procedure where good organ function is quickly restored by the transplant and, typically, the patient can return to an active lifestyle. Unfortunately, the human immune system reacts strongly against allogeneic tissues, and will destroy a transplanted organ within days or weeks after transplantation if the immune system is not depleted or inhibited by immunosuppressive drugs. Near elimination of the immune system through non-specific [1] or specific depletive therapies [2] prior to transplantation is one strategy that is effective at preventing early reactions to the graft, and in a few cases in establishing tolerance-promoting chimerism [3,4]. But these treatments are harsh with significant side effects, and donor tolerance is not assured after the immune system recovers. Thus, the current standard of care for transplant recipients involves the use of general immunosuppressive drugs that reduce graft destruction. The downside of their use is that the whole immune system is impaired, often causing a myriad of side-effects (for example, toxicity, infection, and malignancy), and chronic rejection remains a long-term problem. The lack of improvement in 10-year organ survival rates over the past decades for renal [5] and liver [6] transplantation highlight the need for new therapeutic approaches to prevent organ destruction.

## Cell therapy: *The ONE Study* focus

*The ONE Study* is the acronym for a large scale collaborative project funded by the European Community’s FP7 [7]. The project is entitled: “A Unified Approach to Evaluating Cellular Immunotherapy in Solid Organ Transplantation” and involves scientists from Germany, France, Italy, the United Kingdom and the United States with the aim of improving treatment and the overall quality of life for kidney transplant patients through cell therapy. The goal of the project is to develop and test an array of novel manufactured cell therapy products that have promise to reduce a transplant patient’s life-long dependency on immunosuppressive drugs. In principle, the hypothesis behind cell therapy in the context of organ transplantation is that treating recipients with a concentrated dose of well-defined regulatory immune cells near the time of transplantation will trigger a self-sustaining immune regulation that establishes a level of functional protection against allograft destruction. This concept is fundamentally different from other modes of treatment for transplant recipients since the therapy supplements, or ‘adds-in’, a functional cellular component of the immune system that is believed capable of precipitating and establishing a protective immunological balance favoring allograft survival. In contrast, all other current forms of therapy seek to take away immune function through depletion of classes of detrimental immune cells, or through blockage of signals in these cells required for immune reactions; this form of ‘functionally inhibitory’ therapy primarily causes a general suppression of the immune system.

*The ONE Study* consortium explores the feasibility and potential of cell therapy in organ transplantation by bringing together experts to test different regulatory cell products composed of specific T cell (T regulatory cell, Treg and T regulatory type 1 cell, Tr1 cell), dendritic cell and macrophage subpopulations. While the primary objective of the study is to perform a stringent side-by-side comparison of these cell products using a single (ONE) standardized trial protocol, a secondary aim is to use this unique cooperative opportunity to experimentally examine the immunoregulatory characteristics of the cell products to be tested.

## Monocyte-derived regulatory cell populations

To address the issue of comparing and contrasting suppressive cell populations with cell therapy potential, a series of investigative workshops have been planned within the context of *The ONE Study*. The first of these workshops has already taken place this year with an emphasis exclusively on producing and comparing suppressive populations of cells derived from monocytes, namely tolerogenic dendritic cells (tol DCs) [8], myeloid-derived suppressor cells (MDSCs) [9], IL-10-induced DCs (DC-10 cells) [10] and regulatory macrophages (M regs) [11,12]. A central premise of this workshop is that each cell type is produced side-by-side in a single laboratory from the same leucapheresis products, thus eliminating differences attributable to cell sources and inter-laboratory conditions. This type of collaborative effort is only possible through a large-scale integrating grant such as *The ONE Study*. While the final results of this first workshop are still pending, the assembled group of workshop participants decided it would be informative to perform a separate review of each of the monocyte-derived regulatory cell populations under investigation.

In the current issue of *Transplantation Research*, workshop participants present literature and their perspectives on the specific cell population that they produced for comparison. It is important to keep in mind that the workshop investigators are studying the different monocyte-derived regulatory cells more from a cell production standpoint, versus taking a view towards normal physiological development. Indeed, the *in vivo* temporal and complex signals directing a blood monocyte or tissue macrophage to become activated, to take on professional antigen-presenting functions, to migrate to lymphoid structures, or to become suppressive, are not fully understood. However, the workshop participants primarily aim to distinguish or show similarities between suppressive monocyte-derived cell populations that can be generated under defined *in vitro* conditions. This is an important undertaking since cell therapy applications demand an *ex vivo* cultivation phase to manufacture a homogenous and reproducible cell product.

For comparison, six cell populations were derived from blood monocytes by investigators of the workshop group: (1) tol DCs, (2) DC-10 cells, (3) rapamycin-conditioned DCs (rapa DCs), (4) MDSCs, (5) M regs and (6) monocytes conditioned with mesenchymal stem cells (MSCs). The variety of stimuli that are considered for cell production is especially evident with tol DCs. The workshop group concentrated on tol DCs generated under conditions including stimulation with low concentrations of GM-CSF [13], and with combinations of GM-CSF, IL-4 and IL-10 (DC-10 cells [14]) or rapamycin (rapa DCs [15]). Besides comparing the characteristics (gene expression, phenotype, function, *etc.*) of these closely related cells to each other, they are comparable to cells such as MDSCs, which can be generated under similar conditions, but in the presence of prostaglandin E_2_[16] or other stimuli. Interestingly, to further show the potential overlapping properties of monocyte-derived suppressive cells, recent evidence suggests that MSCs mediate their immunosuppressive effect through induction of an auxiliary macrophage via IL-10 or prostaglandin E_2_[17]; whether these conditioned macrophages are unique or similar to other suppressive macrophages is a question to be addressed through the workshop. Finally, our own group in Regensburg has been actively studying the suppressive properties of monocyte-derived cells generated through M-CSF-induced maturation in combination with late *ex vivo* interferon gamma stimulation (M regs [18]). While M regs have a distinct phenotype compared to other known suppressive macrophages [12], and have already been used in a limited number of transplant recipients [11], it remains unclear how these cells compare phenotypically and functionally to *ex vivo* generated tol DCs or MDSCs; *The ONE Study* workshop also seeks to answer this question.

In preparing to assess the results of the first *ONE Study* workshop on monocyte-derived suppressor cells, the involved investigators deemed it useful to review the present literature on tolerogenic DCs and macrophage/myeloid derived suppressor cell populations. The authors of the reviews consider the similarities and differences of these closely related cell populations, and suggest how they may best be applied as a cell therapy meant to reduce organ transplant rejection. Indeed, cell therapy is entering a critical early phase of testing in transplantation, mandating that we take the necessary steps to determine which cell populations are most adaptable and effective for this purpose. The reviews in this issue of *Transplantation Research* offer a view into the future of cell therapy involving suppressive cells derived from readily available circulating monocytes.
